# The Evolving Legal Standard for Medical Malpractice: Implications for Otolaryngology–Head and Neck Surgery

**DOI:** 10.1002/ohn.1326

**Published:** 2025-05-28

**Authors:** Shiven Sharma, Margo K. McKenna, Michael J. Brenner

**Affiliations:** ^1^ Department of Otolaryngology Icahn School of Medicine at Mount Sinai New York New York USA; ^2^ University of Rochester Medical Center/Golisano Children's Hospital Rochester New York USA; ^3^ Department of Otolaryngology–Head and Neck Surgery University of Michigan Medical School Ann Arbor Michigan USA

**Keywords:** clinical practice guidelines, defensive medicine, evidence‐based medicine, facial plastic surgery, head and neck oncology, informed consent, malpractice litigation, medical malpractice, Otolaryngology–Head and Neck Surgery, patient safety, patient‐centered care, patient‐centric care, physician‐patient communication, rhinology

## Abstract

The recent revision of medical malpractice standards by the American Law Institute (ALI) represents a shift from a customary practice‐based model to an evidence‐based approach that emphasizes reasonable and patient‐centric medical care. This transition acknowledges prevailing medical customs but prioritizes guidelines and contemporary scientific knowledge in determining the reasonable standard of care. While adherence to clinical guidelines may serve as a defense against malpractice claims, approaches that differ from them are not necessarily considered negligent, allowing for individualized patient care. Given otolaryngology's leadership in developing and implementing clinical practice guidelines, this legal evolution has significant implications for patient safety, physician autonomy, and malpractice risk. Guidelines act as a safeguard against defensive medicine, minimizing unnecessary tests and bolstering the use of evidence‐based interventions with established risks. This commentary explores how the new standard aligns with evidence‐based medicine, its potential influence on defensive medicine, and its broader ramifications for the otolaryngology community.

Medical malpractice law in the United States has traditionally relied on customary practices within a medical specialty to define the standard of care.^1^ The American Law Institute (ALI), an independent organization that restates legal principles, has historically reinforced this approach. While ALI restatements are not legally binding, they are frequently cited by state courts in judicial decisions. Recently, however, the ALI approved the Restatement (Third) of Torts: Medical Malpractice, which marks a shift toward a more patient‐centric and evidence‐based approach.^2^ The change, summarized in a recent *JAMA* article by Aaron et al., represents a major legal evolution that will gradually reshape how courts evaluate medical negligence.^3^

Otolaryngology–Head and Neck Surgery (OHNS), as a leader in clinical practice guideline (CPG) development, stands at the forefront of this legal transformation. This commentary explores the implications of this shift, highlighting implications for promoting evidence‐based practice, reducing defensive medicine, and improving physician‐patient communication.

## A Shift From Custom to Evidence

The traditional malpractice standard has long emphasized what is customary among competent practitioners. However, the ALI's revised framework shifts toward a more nuanced assessment of reasonable medical care.^3^ The new standard defines reasonable care as the skill and knowledge of competent providers in similar circumstances, enabling courts to consider contemporary evidence‐based guidelines and best practices in malpractice claims.^3^

For OHNS, where CPGs are widely used and rigorously developed,^4^ this evolution in malpractice standards reinforces the role of evidence‐based decision‐making in patient care. The ALI's revised framework reflects precedent evident in some state judicial decisions, and future rulings will increasingly turn on whether prevailing practices align with scientific evidence.^3^

## Guidelines Not a Double‐Edged Sword

The new standard recognizes adherence to guidelines as a defense against malpractice claims^3^; however, care that differs from guidelines is not necessarily inculpatory—that is, care that deviates from guidelines can be appropriate and does not necessarily constitute negligence.^3^ This distinction is crucial in OHNS, where individual patient context can provide a rationale for care that departs from guideline‐based recommendations. For example, a surgeon might reasonably deviate from a guideline‐recommended treatment approach based on patient‐specific anatomical, functional, or comorbid considerations. Courts will now assess whether such deviations are justifiable based on sound clinical reasoning tailored to the case rather than assuming negligence.

## Impact on Defensive Medicine

This evolving malpractice standard could support a reduction in defensive medicine— those practices where physicians order unnecessary tests or procedures primarily to mitigate perceived legal risk, sometimes to the detriment of patient‐centric care.^3^, ^5^ The ALI's new standard, by providing greater clarity, could reduce defensive medicine. It parallels initiatives like Choosing Wisely, which aim to curb unnecessary care by promoting guideline‐based recommendations and informed surgeon–patient conversations.

Most CPGs include quality improvement statements to help reduce unnecessary testing or treatment (eg, “Clinicians should not obtain radiographic imaging for patients who meet diagnostic criteria for acute rhinosinusitis, unless a complication or alternative diagnosis is suspected” or recent guidance on age‐related hearing loss).^4^, ^6^, ^7^ These guidelines lend further support toward a move away from defensive medicine. Otolaryngologists can exercise sound judgment without fear that deviation from customary, but outdated, practices might be construed as malpractice. Additionally, this framework could encourage further investment in developing high‐quality, evidence‐based guidelines, knowing that adherence can serve as a legal safeguard.

## Recent Malpractice Trends in Otolaryngology

A demographic analysis of malpractice claims from 2018 to 2024 identified 79 court proceedings and indicated that head and neck oncology was the most sued subspecialty (32.5%), followed by rhinology (15.6%). While most jury verdicts (87.7%) favored the defendant, the average verdict payout for the plaintiff was $7.43 million.^8^ These cases may represent a small fraction of lawsuits, as most are likely settled outside the courtroom.^9^ An internal review of captive insurance claims from 2000 to 2020 at one tertiary care health system found 28 claims, with head and neck surgery being the most frequently implicated subspecialty (32.1%).^10^ The most common causes of litigation included surgical performance (35.7%) and failure to diagnose (28.6%).^9^ The shift in ALI standard could support progress in evidence‐based care.

## Physician‐Patient Communication and Informed Consent

The ALI's revisions also extend to the legal expectations surrounding physician‐patient communication. Physicians’ representations of their skills, experience, and available treatment options now play a role in assessing the reasonableness of care.^2^ In OHNS, where procedures range from routine interventions to complex oncologic and reconstructive surgeries, the implications are significant. Ensuring that patients understand the available options, the rationale for treatment, complexity of procedures, surgeon experience, potential complications, and evidence‐based alternatives can enhance patient care and reduce liability risk.

## Recommendations for Otolaryngology Stakeholders

Considering this legal change, professional societies should take a leading role in developing and maintaining clinical guidelines through transparent, methodologically rigorous processes, and ensure those guidelines are regularly updated and effectively disseminated. Meanwhile, medical malpractice insurers should offer incentives for participation in continuing education and update policy language to reflect evolving standards. Furthermore, risk management personnel should focus on promoting strong documentation practices, integrating guidelines into clinical workflows, and supporting clinician education to reduce liability and improve compliance.

## Conclusion

The ALI's revised approach to medical malpractice law marks a significant shift in how courts evaluate medical negligence, favoring a more evidence‐based and patient‐centric framework. By aligning practice with evidence‐based guidelines, fostering effective physician‐patient communication, and mitigating defensive medicine, the OHNS community can proactively adapt to this new era of malpractice law.

## Author Contributions

**Shiven Sharma**, conception, design, and drafting of the work, acquisition, analysis, and interpretation of data for the work; critically revising the work for important intellectual content; approval of the final submission; agreement to be accountable for all aspects of the work. **Margo K. McKenna**, substantial contribution to analysis and interpretation of data for the work; critically revising the work for important intellectual content; approval of the final submission; agreement to be accountable for all aspects of the work. **Michael J. Brenner**, conception, design, and drafting of the work, acquisition, analysis, and interpretation of data for the work; critically revising the work for important intellectual content; approval of the final submission; agreement to be accountable for all aspects of the work.

## Disclosures

### Competing interests

None.

### Funding source

None.
